# Inertial sensing of water content in tumor spheroids

**DOI:** 10.1126/sciadv.aeb1451

**Published:** 2026-04-03

**Authors:** Georgios Katsikis, Jennifer C. Yoon, Thomas R. Usherwood, Seth Malinowski, Jiaquan Yu, Chuyi Chen, Sukbom Son, Julie L. Sutton, Keith L. Ligon, Jungchul Lee, Teemu P. Miettinen, Scott R. Manalis

**Affiliations:** ^1^ Koch Institute for Integrative Cancer Research, Massachusetts Institute of Technology, Cambridge, MA 02139, USA.; ^2^ Harvard-MIT Department of Health Sciences and Technology, Institute for Medical Engineering and Science, Massachusetts Institute of Technology, Cambridge, MA 02139, USA.; ^3^ Department of Pathology, Dana-Farber Cancer Institute, Harvard Medical School, Boston, MA 02115, USA.; ^4^ Department of Mechanical Engineering, Korea Advanced Institute of Science and Technology, 291 Daehak-ro, Yuseong-gu, Daejeon 34141, South Korea.; ^5^ Center for Patient-Derived Models, Dana-Farber Cancer Institute, Boston, MA 02115, USA.; ^6^ Department of Pathology, Brigham and Women’s Hospital, Harvard Medical School, Boston, MA 02115, USA.; ^7^ Department of Mechanical Engineering, Massachusetts Institute of Technology, Cambridge, MA 02139, USA.; ^8^ Department of Biological Engineering, Massachusetts Institute of Technology, Cambridge, MA 02139, USA.

## Abstract

Cellular water content governs the concentration of all biomolecules inside a cell, thereby influencing the physical and functional properties of the cell. However, measurements of water content in physiologically relevant cell culture models remain largely unavailable, particularly in three-dimensional (3D) models such as tumor spheroids and organoids. Here, we achieve such measurements using an industrial-grade capillary steel tube. The steel tube functions as a mechanical resonator that inertially senses the buoyant mass of particles. For microgram-scale particles ≥ 400 micrometers in diameter, we achieve <1% precision error in buoyant mass with a 5-minute acquisition interval. By sequentially measuring the buoyant mass of individual, patient-derived glioblastoma tumor spheroids derived from patients with glioblastoma in media of different densities and cell permeabilities, we determine the absolute and fractional (volume/volume) water content of the spheroids, along with their dry mass, volume, and density properties. We achieve ~0.5% precision error in fractional water content with a throughput of three spheroids per hour. This enables us to detect both interspheroid heterogeneity in fractional water content and acute responses to kinase inhibition. Overall, we establish a simple and accessible technique for quantifying water content in living 3D cell culture models, opening previously unexplored avenues for studying biophysical regulation in multicellular systems.

## Teaser

A vibrating steel tube measures water content in individual living tumor spheroids.

## INTRODUCTION

Almost all known organisms are composed primarily of water. Within individual cells, water content defines the concentration of biomolecules, determines the extent of molecular crowding, and influences physical properties such as mechanical stiffness (*1*, *2*). Processes such as intracellular transport, protein synthesis, cell cycle progression, and cell divisions have all been shown to respond to, or be coupled with, changes in intracellular water content and crowding (*3*–*17*). These effects are relevant in all model systems, including multicellular structures. For example, stem cell self-renewal within organoids is sensitive to changes in cellular water content (*1*, *6*). Because water content is partially regulated by cell-matrix adhesion (*1*, *11*, *18*), mechanisms of water homeostasis may differ between isolated cells and cells embedded within multicellular environments. However, measurement of water content in multicellular structures remains elusive, and current research relies on osmotic perturbations of water content without direct water content quantifications. Consequently, the extent to which multicellular models display water content homeostasis and how substantially such models change their water content in response to cell state changes remains largely unexplored.

Tumor organoids and spheroids, such as neurospheres derived from brain tumors, are three-dimensional (3D), multicellular structures composed of cancer cells. These next-generation 3D cell culture models have emerged as powerful and tractable alternatives to classical in vitro 2D cell lines reducing dependence on animal models. However, tools to measure properties and drug responses of tumor spheroids remain limited. Measuring their biophysical properties can provide valuable insights, as tumor spheroid size has been shown to reflect both drug responsiveness and susceptibility to immune-mediated antitumor effects (*19*–*21*). More broadly, biophysical heterogeneity among tumor spheroid and organoid poses a major challenge to reproducibility in preclinical research (*22*, *23*). Currently, biophysical characterization of these systems relies primarily on imaging-based approaches (*22*). For example, measurements of spheroid diameter and sinking velocity have been used to estimate spheroid mass and density (*24*), and stimulated Raman scattering microscopy has been used to assess spheroid dry mass composition (*25*–*27*). In addition, electrical impedance spectroscopy can be used to assess spheroid size (*28*). However, no existing technique enables direct measurement of water content in tumor spheroids or other multicellular model systems.

Here, we developed an approach for directly quantifying the water content of large (~500-μm diameter) tumor spheroids. Our approach uses an inertial resonator sensor that determines sample mass by detecting shifts in its resonant frequency induced by the presence of the sample. These sensors have been previously applied across a wide range of biological scales, including viruses (*29*) bacteria (*30*, *31*), yeast (*32*, *33*), phytoplankton (*34*), protozoa (*35*), and mammalian cells ranging from 4 to 50 μm in diameter (*9*, *36*, *37*), as well as nonbiological particles smaller than 50 μm in diameter (*37*–*39*). However, conventional resonator sensors, typically based on microelectromechanical systems fabricated from silicon in a cleanroom (*40*), are constrained by their small dimensions and cannot accommodate larger biological models. Conversely, larger resonator sensors constructed from glass can accommodate greater sample sizes (*41*, *42*), but they generally lack the sensitivity required to resolve the mass of samples in the tumor spheroid size range (300 to 600 μm). Our sensor bridges this gap by combining a suitable form factor for submillimeter biological samples with the high sensitivity necessary for accurate analysis of mass, density, and water content.

Our sensor measures the buoyant mass ($m_{b}$) of a sample, as it flows through a resonating steel tube. We defined buoyant mass as

$$m_{b}=V_{\text{total}}\;\left(\rho_{\text{total}}-\rho_{\text{fluid}}\right)$$ (1)

where $V_{\text{total}}$ is the volume of the sample and $\rho_{\text{total}}$ and $\rho_{\text{fluid}}$ are the densities of the sample and the measurement fluid, respectively. By sequentially measuring the buoyant mass of the same sample in multiple fluids with distinct densities and cell permeabilities, we can determine the total volume and density (*9*, *33*, *42*, *43*) and the dry volume and density of the sample (*14*, *32*). This enables the quantification of the absolute and fractional water content of individual samples, such as tumor spheroids.

Although similar principles have been applied to infer the average water content in populations of single cells (*17*, *34*), this has not previously been achieved at the level of individual 3D cell culture models. Using our method, we reveal previously inaccessible heterogeneity in fractional water content across patient-derived glioblastoma (GBM) tumor spheroids and demonstrate that water content can be modulated by pharmacological perturbation. These findings illustrate the potential of our approach to resolve biologically meaningful variations in water content at single-spheroid resolution.

## RESULTS

### Steel tube vibrates at higher-order resonant modes

**Fig. 1.**
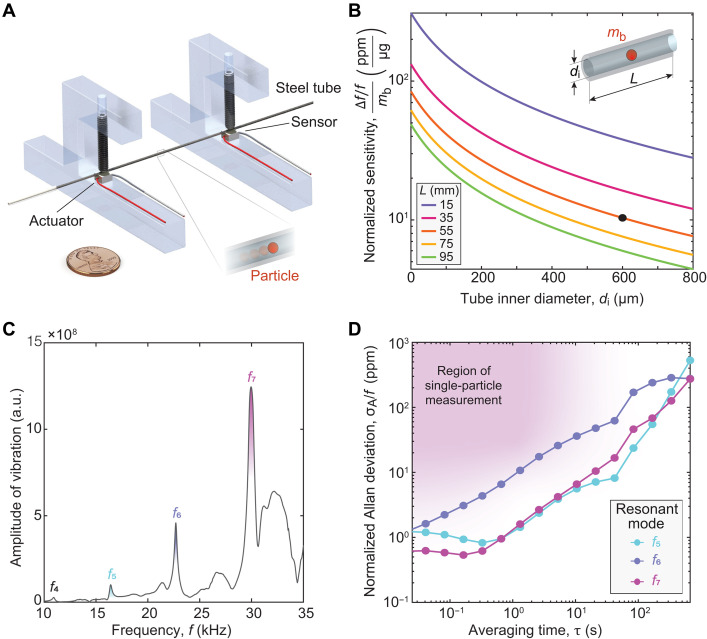
A steel tube resonator. (**A**) Schematic of a steel tube actuated to vibrate at its resonant frequencies. A United States penny (diameter: 19.05 mm) denotes the length scale. (**B**) Theoretical prediction of normalized mass sensitivity as a function of length *L* and inner diameter $d_{i}$ of tube. We designed the tube to yield normalized sensitivity (~10 ppm/μg), which suffices for spheroids (~400- to 500-μm diameter) of microgram-scale buoyant mass *m*_b_ (note S1). The black dot denotes our selected dimensions. (**C**) Experimental amplitude of vibration versus frequency, displaying four local maxima, which correspond to the fourth to seventh modes of resonant frequencies. (**D**) Allan deviation, σ_A_, of noise, normalized by baseline frequency at resonant modes excited by a phase-locked loop (PLL) control. The purple area indicates the region of measuring single particles with transit times on the order of seconds through the tube and well above the optimal noise for the seventh mode.

We deployed an industrial-grade steel tube with a 600-μm inner diameter ($d_{i}$) as the core sensing element. Given the tube’s straight geometry and open ends, we implemented a double-clamped configuration to support vibration (Fig. 1A and fig. S1A). We selected stainless steel (SUS304) for its biocompatibility, high compressive strength (~200 MPa), and standardized manufacturing processes (*44*). We also defined the dimensions of the tube for practical assembly while maintaining the required sensitivity for the measurement based on theoretical predictions (note S1). In particular, we designed the tube to yield normalized sensitivity of 10 parts per million (ppm)/μg (e.g., a signal of 0.3 Hz/μg for baseline frequency of 30 kHz), which suffices for spheroids (~400- to 500-μm diameter) of microgram-scale buoyant mass (Fig. 1B). We mounted each end of the tube using a screw onto a piezoelectric element with one designated for actuation and the other for sensing. The tube’s ends were connected via flexible tubing to temperature-controlled, pressurized medium vials, allowing particles to be flown back and forth by modulating the pressure between vials. A T-junction in the flow path was connected to a syringe, enabling isolation of individual spheroids within a small volume and a complete exchange of the fluid inside the resonator (fig. S1A).

To confirm the tube’s behavior as a resonator, we vibrated it in air across a range of frequencies and observed distinct amplitude peaks corresponding to its fourth through seventh bending resonant modes (Fig. 1C). These peaks agreed with theoretical predictions in the kilohertz range (fig. S1B). We then used a phase-locked loop (PLL) control to selectively excite each one of these resonant modes (*45*). The seventh mode exhibited the lowest mass-equivalent noise (*m*_σ_ ~ 0.5 ppm or 0.05 μg), as determined via Allan deviation analysis for single-particle transits lasting several seconds (Fig. 1D and fig. S1C). We used the seventh resonant mode (quality factor *Q* = 410) for all experiments from here on.

### Steel tube measures microgram-scale particles

**Fig. 2.**
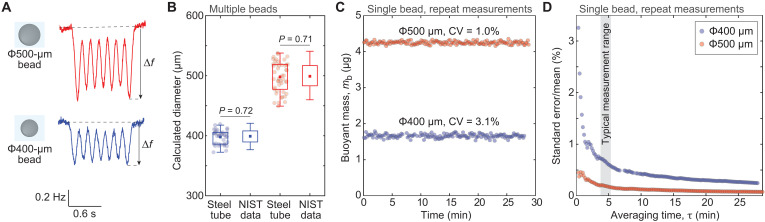
High-precision buoyant mass measurements of microgram-scale particles. (**A**) Representative signals of frequency change when flowing 500- and 400-μm polystyrene particles through the steel tube vibrating at the seventh mode with PLL control (Materials and Methods). Δ𝑓 denotes the maximum absolute change of frequency. (**B**) Steel tube measurements of Δ𝑓 signals for 500- and 400-μm particles converted to diameter using the known density of particles and fluid. Particle reference size [National Institute of Standards and Technology (NIST) data] is shown for comparison. Individual dots depict single beads (*n* = 34 and 50). *P* values obtained using *F* test. (**C**) Repeated Δ𝑓 measurements of a single particle, displayed as the buoyant mass. Data are shown for a 500-μm and a 400-μm particle (red and blue, respectively). *n* = 170 and 161 repeat measurements. CV represents the coefficient of variation. (**D**) Repeated measurement precision (SEM, normalized to %) as a function of measurement acquisition time for 500- and 400-μm particles. The typical time range for repeated measurements in tumor spheroids is shown in gray.

We evaluated the performance of our particle-measurement system using 400- and 500-μm polystyrene beads. As these beads passed through the vibrating tube, the seventh-mode frequency response with PLL control behaved as expected (Fig. 2A) (*46*). We quantified the resulting frequency shifts (Δf) and calibrated our measurements by computing the tube’s effective mass ($m_{\text{eff}}$) during bead transits (Materials and Methods). The experimentally determined sensitivity (~9 ppm/ug) agreed with theoretical predictions to within 10% (Fig. 1B). Using this calibration, we calculated $m_{\text{eff}}$ for both bead sizes and confirmed that the measured size distributions matched the variability reported by the supplier (Fig. 2B). We further evaluated the calibration accuracy with independent bead measurements and calibration procedures (note S2).

We then examined the precision of our system using repeat measurements of a single bead flowing back and forth through the steel tube. This revealed a coefficient of variation (CV) of 1 to 3% for the buoyant mass measurements, depending on bead size (Fig. 2C). Using the same data, we quantified our measurement precision as a function of acquisition time (Fig. 2D). Opting for 5 min of measurement (~25 repeats) yielded a precision error < 1% for both 400- and 500-μm beads. We used a comparable acquisition time in all experiments with tumor spheroids. Last, we examined the stability of our system during the repeated measurements of a single bead. We did not observe any correlation between the measured bead size and time (*P* = 0.48 and *P* = 0.49 for 400- and 500-μm beads, respectively). The measurement precision remained similar throughout the single bead measurement (fig. S2A), and the measurement noise did not display any repeating patterns, as indicated by the lack of autocorrelation between consecutive bead measurements (fig. S2B).

### Quantification of absolute and fractional water content in tumor spheroids

**Fig. 3.**
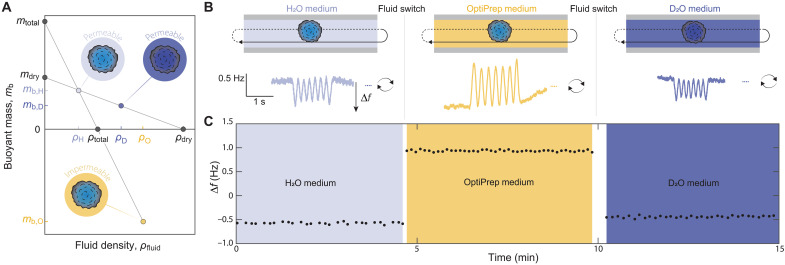
Weighing tumor spheroids in multiple fluids to determine their biophysical properties. (**A**) Graphical solution of methodology to calculate biophysical properties in tumor spheroids. Total mass ($m_{\text{total}}$) and density ($\rho_{\text{total}}$) are calculated by combining the measurements in H_2_O and OptiPrep media using Eq. 1. Dry mass ($m_{\text{dry}}$) and dry density ($\rho_{\text{dry}}$) are calculated by combining the measurements in H_2_O and deuterium oxide (D_2_O) media using Eq. 2. The densities $\rho_{H},\rho_{D},\rho_{O}$ respectively refer to H_2_O, D_2_O, and OptiPrep media. These measurements allow us to derive dry volume and total volume, which are used to calculate fractional water content, $r_{w}$, using Eq. 3. (**B**) Schematic of a single tumor spheroid sequentially measured in three different fluids (top). In each fluid, the sample flows back and forth through the resonating tube to obtain multiple buoyant mass measurements (Δ𝑓). Examples of experimental signals are shown at the bottom. (**C**) An example of Δ𝑓 over time for a single tumor spheroid in the three measurement fluids.

Having established buoyant mass measurements for microgram-scale particles, we next wanted to use our measurements to quantify the water content of individual tumor spheroids by measuring the buoyant mass of the same tumor spheroid in different fluids. First, we measured buoyant mass in normal culture medium and in high-density culture medium that contains 35% OptiPrep, which is a cell-impermeable, isotonic density gradient component. With knowledge of the density of both fluids, the two buoyant mass measurements allow us to determine the total volume, mass, and density of the tumor spheroid using Eq. 1 (Fig. 3A) (*9*, *33*, *42*, *43*). Notably, these volume measurements are independent of the sample’s shape, thus achieving a high precision even on irregularly shaped tumor spheroids (*43*).

Then, we measured the same tumor spheroid in medium made in heavy water, deuterium oxide (D_2_O), which has a higher density than H_2_O. However, unlike OptiPrep, D_2_O rapidly enters the cells and replaces intracellular H_2_O (*14*, *32*, *47*). As the intracellular and extracellular water now have equal densities in the H_2_O- and D_2_O-based media, the measurements are only sensitive to the dry components of the tumor spheroid. We can therefore consider buoyant mass as

$$m_{b}=V_{\text{dry}}\;\left(\rho_{\text{dry}}-\rho_{\text{fluid}}\right)$$ (2)

where $V_{\text{dry}}$ is the dry volume, $\rho_{\text{dry}}$ is the density of the dry mass (i.e., dry mass over dry volume), and $\rho_{\text{fluid}}$ is the density of the measurement fluid (*14*, *32*). With knowledge of the density of both H_2_O and D_2_O, we can combine the two buoyant mass measurements to determine the dry volume, dry mass, and dry density of the tumor spheroid using Eq. 2. Comparing the total volume ($V_{\text{total}}$) and dry volume ($V_{\text{dry}}$) from Eqs. 1 and 2, we can then calculate both the absolute and fractional ($r_{w}$) [% (v/v)] water content for individual tumor spheroids

$$r_{w}=\frac{V_{\text{total}}-V_{\text{dry}}}{V_{\text{total}}}$$ (3)

The calculation of tumor spheroid’s total density, dry density, and fractional water content is largely independent of our system calibration, as the calibration factors cancel out (note S2). To implement these measurements in tumor spheroids, we used patient-derived GBM tumor spheroids (neurospheres) as a model system (*48*). Because OptiPrep has limited permeability to extracellular spaces within the spheroids, the measured water content reflects both intra- and extracellular water. Each spheroid was measured ~30 times over ~5 min in each medium (Fig. 3, B and C). For medium changes, we transferred the spheroid into a syringe containing a small volume of the current medium (fig. S1A), while the system was flushed with the new medium. Because our measurements are highly sensitive to the density of the fluid inside the steel tube, medium changes and imperfect mixing introduced fluctuations in the measurement baseline (fig. S3A). Despite this, we obtained stable, repeatable measurements of spheroid buoyant mass (Fig. 3C), with a precision error of less than 1% in all media (fig. S2C). Repeated measurements showed no systematic change in spheroid mass, indicating sufficient structural integrity of the measured spheroids throughout the process. Overall, our approach enabled quantification of each spheroid’s water content within ~15 min. We also assessed whether brief exposure to D_2_O affected long-term spheroid viability (fig. S4A) and observed no detectable impact (fig. S4B).

### Detection of heterogeneity in water content between individual tumor spheroids

To assess the biological heterogeneity of tumor spheroids, we examined the precision of our water content measurement. This is critical, as observed heterogeneity could arise from measurement noise alone. As there are no standard particles with fixed water content that could be used to define our measurement precision, we pursued two approaches.

**Fig. 4.**
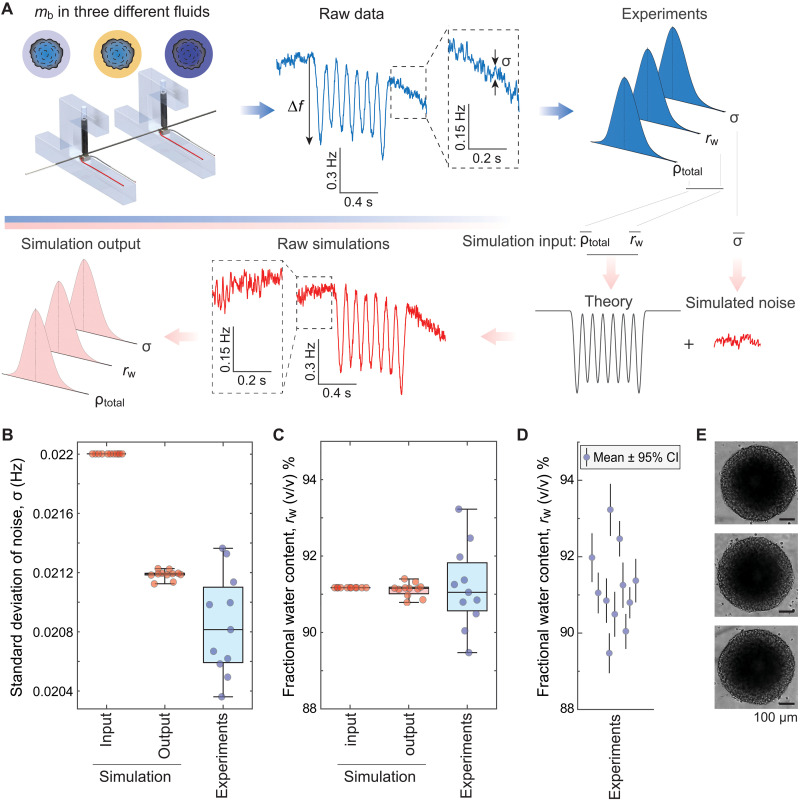
Detection of heterogeneity in water content of tumor spheroids. (**A**) Methodology of water content measurement simulations. Experiments with tumor spheroids yield experimental traces (blue) where baseline noise magnitude (σ) and frequency change (Δ𝑓) are quantified. Taking the means of fractional water content (${\bar{r}}_{w}$), total density (${\bar{\rho }}_{\text{total}}$), and noise magnitude ($\bar{\sigma }$) as simulation inputs, we combined theoretical frequency changes and simulated noise to generate simulated raw data (red) that mimic the experimental data. These simulated data are analyzed identically to the experiments to yield simulation output values for $r_{w}$, ρ_total_, and σ. The simulated data assume similar heterogeneity in tumor spheroid sizes as observed in the experiments but no heterogeneity in tumor spheroid $r_{w}$ or $\rho_{\text{total}}$. (**B** and **C**) Comparison of σ (B) and $r_{w}$ (C) between simulation (red) and experiments (blue). Each dot refers to a single tumor spheroid (*n* = 11 for all conditions). (**D**) Measured $r_{w}$, and the 95% confidence interval (CI) of the measurements for single tumor spheroids. The error bars were determined on the basis of $m_{b}$ measurement error propagation. (**E**) Representative bright-field images of three tumor spheroids.

First, we assessed measurement precision in silico by simulating buoyant mass signals with noise characteristics matched to those observed experimentally (Fig. 4A and Materials and Methods). To quantify low-frequency baseline fluctuations in the experiments, we fit a third-order polynomial to the baseline regions immediately before and after each peak (fig. S3, B to D). After removing these baseline fluctuations with a high-pass Butterworth filter, we characterized the remaining noise by its SD (σ) (fig. S5A). In the simulations, we set $r_{w}$ and $\rho_{\text{total}}$ equal to experimental means and varied only the spheroid size to reproduce the distribution observed experimentally (Fig. 4A). Thus, the simulated spheroids constituted a homogeneous population in wet and dry biophysical properties, differing only in size. To confirm that the simulations faithfully reproduced experimental noise conditions, we reanalyzed the noise magnitude in the simulated data and found that the simulated noise corresponded to the upper (>80th) percentile of experimental noise distribution (Fig. 4B).

Our simulations revealed a precision error of ~0.4% [95% confidence interval (CI)] in the fractional water content, $r_{w}$ (Fig. 4C). The experimental tumor spheroid data showed ~6-fold more fractional water content heterogeneity between individual spheroids than our simulations (*P* < 0.0001; *F* test of equality of variances). Thus, our measurements can, in principle, resolve the water content heterogeneity between similar-sized individual tumor spheroids. Similarly, we determined that the precision errors of total and dry densities, respectively, are ~0.0001 and ~ 0.016 g/ml (95% CI) (fig. S6). Our experiments showed more heterogeneity for total and dry density, suggesting that heterogeneity is biological.

To ensure that our fractional water content measurement precision is not biased by specific noise characteristics, we simulated tumor spheroids using different types of colored noise (slope a=0−2 of power spectral density of noise). These simulations showed that the fractional water content precision is largely insensitive to the slope a (fig. S5B). However, when the noise magnitude (σ) was increased well beyond levels observed in experiments, the noise slopes began to affect precision (fig. S5C), highlighting the importance of maintaining high measurement quality. In addition, we confirmed that the noise magnitude observed for each tumor spheroid did not account for the observed heterogeneity in water content (fig. S5D).

Second, we assessed the precision of our fractional water content ($r_{w}$) determination by quantifying and propagating experimental error of our measurements in each fluid. By combining Eqs. 1 to 3, we obtained an analytical expression for $r_{w}$ in terms of the directly measured buoyant masses and performed error propagation analysis (note S3). This analysis yielded an error of ~0.55% (95% CI). This is in close agreement with simulation-based estimates (~0.4%), indicating that our approach is sufficiently sensitive to resolve intraspheroid heterogeneity in $r_{w}$ (Fig. 4D). We confirmed this statistically using a weighted-residual chi-square test (*P* = 2 × 10^−28^), which rejected the hypothesis that all spheroids have identical $r_{w}$. Pairwise Welch-Satterthwaite *t* tests further showed that most spheroids differed significantly from one another in $r_{w}$ (*P* < 0.05 for the majority of comparisons; note S3). This heterogeneity was not apparent from bright-field imaging of the tumor spheroids (Fig. 4E).

Last, we evaluated whether incomplete intracellular water exchange upon transfer to the D_2_O-based medium could bias our water content measurements. Because replacement of H_2_O with D_2_O increases buoyant mass in the D_2_O medium, incomplete exchange would be expected to produce a systematic rise in buoyant mass over consecutive measurements. However, no such increase was observed (fig. S7A). We further tested the sensitivity of our $r_{w}$ estimates by recalculating $r_{w}$ after excluding the earliest D_2_O measurements, effectively assuming incomplete H_2_O to D_2_O exchange. This omission had only a minimal effect on the resulting $r_{w}$ values (fig. S7B). Together, these results indicate that incomplete water exchange does not measurably bias our water content measurements.

### Staurosporine induces an acute loss of water in tumor spheroids

**Fig. 5.**
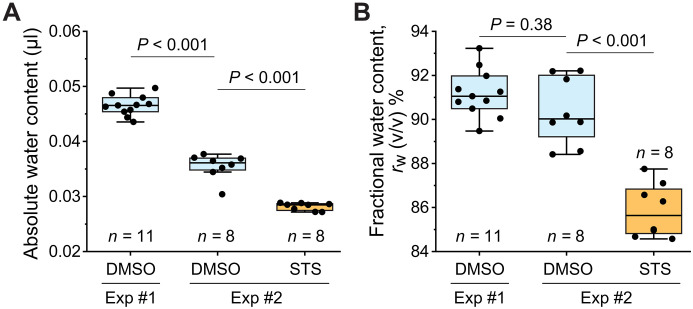
Staurosporine induces an acute loss of water in tumor spheroids. (**A**) Absolute water content (volume) and (**B**) fractional water content, $r_{w}$ (%), in individual GBM tumor spheroids in two experiments. Tumor spheroids were treated for 1 hour with DMSO (blue) or 2 μM staurosporine (STS; orange) before measurements. *P* values were obtained using analysis of variance (ANOVA) and Tukey’s post hoc test. Exp, Experiment.

Having established our method for measuring water content with high precision, we next sought to examine perturbations of cellular water content. Staurosporine is a broad-spectrum protein kinase inhibitor that causes apoptosis and an acute loss of intracellular water (*43*, *49*). To examine the effects of staurosporine on tumor spheroids, we treated the tumor spheroids for 1 hour with 2 μM staurosporine or with 0.1% dimethyl sulfoxide (DMSO; vehicle) across two independent experiments. We first observed that the absolute water content of the DMSO-treated (control) spheroids varied between experiments, reflecting overall size differences of the spheroids (Fig. 5A). However, DMSO-treated spheroids in both experiments exhibited similar $r_{w}$ (Fig. 5B), suggesting that tumor spheroids’ fractional water content is independent of their size. In contrast, when we examined staurosporine-treated tumor spheroids, we observed significantly lower $r_{w}$ than in the DMSO-treated spheroids (Fig. 5B). Thus, our method can detect acute changes in water content.

## DISCUSSION

Here, we developed an inertial sensing–based approach to quantify the water content of single tumor spheroids. This method achieves high precision (95% CI of ~0.5%), enabling us to resolve heterogeneity in water content among patient-derived GBM spheroids and to detect acute changes in response to pharmacological perturbation. In addition to quantifying water content, our approach simultaneously measures the mass and density of each sample.

Fractional water content sets the concentration of all biomolecules and thereby influences the biochemical reaction rates and numerous physical properties of the sample. Maintaining appropriate cellular water content is essential for cell fitness, and changes in water content are coupled with cell state, such as the proliferative status (*16*, *17*). However, direct measurement of water content in small 3D model systems has remained elusive. Our approach enables quantitative analysis of how water content varies with growth stage, environmental conditions, and drug perturbations in 3D systems. Because the method is nondestructive, it can be used synergistically with other techniques. For example, while the mechanical resonator quantifies water content and other biophysical properties of irregularly shaped specimens, pairing it with microscopy-based methods could reveal how internal structure and function contribute to these properties (*50*). These multimodal measurements could be used to correlate water content dynamics with metabolic markers, offering insights into how metabolic activity regulates water content in 3D tissue contexts (*51*). Additional insights about the specimen’s physical properties could also be gained by combining impedance dielectric spectroscopy approaches with our approach, either through direct analysis of electrical responses or through models to decouple the effect of interfering elements such as media, channel walls, and electrodes (*52*).

Although demonstrated here with GBM spheroids, our general approach is compatible with a wide range of suspended biological particles. The particle size range can be extended by modifying the resonator tube dimensions. Accordingly, the method is, in principle, applicable to other tumor spheroids, organoids, very large cells (e.g., oocytes and large plankton), and even small multicellular organisms (e.g., tardigrades and *Caenorhabditis elegans*). This ability to study various 3D model systems has clinical potential given the recent emphasis of the National Institutes of Health (NIH) and other funding bodies on the development of nonhuman models, especially in the context of cancer functional precision medicine (*53*). Future work could leverage our approach when studying patient-derived spheroids, correlating water-content heterogeneity with other biological phenotypes and therapeutic responses.

Several limitations should be noted. First, our approach requires samples to be in suspension, which excludes adherent organoid models and matrix-embedded cultures. However, our approach is compatible with current methods using gel-supported cultures, such as alginate droplets, into solution. Second, the samples must be mechanically robust, as repeated flow-based measurements may induce shear stress that could compromise fragile structures. Third, our method relies on the use of OptiPrep to manipulate fluid density. While live cells are impermeable to OptiPrep, extracellular matrix components may permit partial penetration, potentially confounding the distinction between intracellular and extracellular water, especially if samples are maintained prolonged times in OptiPrep. Hence, our measurements reflect total (bulk) water content, without spatial resolution between compartments. Fourth, the selection of steel as a material for the resonator tube limits the signal-to-noise ratio of our measurements in comparison to lighter materials (note S1), thus limiting the size range of measurable samples. Last, our method is relatively low throughput, with a current capacity of ~3 spheroids per hour, limiting its utility for high-throughput screening or large-scale studies.

Despite these limitations, our approach offers a unique capability: It provides quantitative, precise, single-sample measurements of water content in complex 3D biological models—something previously inaccessible with existing techniques. This opens opportunities to investigate water homeostasis and its role in tumor biology, drug responses, and multicellular biophysics across diverse experimental systems.

## MATERIALS AND METHODS

### Fabrication, visualization, and design of devices

The stainless steel capillary tube with an outer diameter of 800 μm and inner diameter of 600 μm (SUS304, Geumyang Materials) is placed on top of two piezo actuators (#TA0505D024W, Thorlabs) that are 5 cm apart. The capillary tubing is clamped with a setscrew that has a soft nylon tip (#AXK117, AXK). Fluorinated Ethylene Propylene tubing (#PB-0201473, IDEX Corporation) is connected at the ends of the capillary tube. The other ends of the tubing are placed inside the glass Wheaton vials, and the temperature of the fluids inside the vials is maintained at ~37°C with the stirring hot plate (#BZA651575, IKA). The 3D model of the system (Fig. 1A) was designed in SOLIDWORKS 2022, and photorealistic effects were added using PhotoView 360. 

### Operation and validation of the system

The two piezo actuators were used where one was used for actuation and the other was used for detection. The piezoelectric actuation signal was amplified with the custom fabricated board amplifier, while the piezoelectric sensor signal by the detection actuator was sent to a voltage preamplifier (#SR560, Stanford Research Systems) that uses a gain of 5000 and the low- and high-pass filter with 3- and 100-kHz cutoff frequencies. Both the actuation and sensor signals were of the order of volts after amplification. The system was run in the PLL of second order with a closed-loop mode where the resonant frequency of the seventh vibration mode was maintained with a bandwidth of 80 Hz. A sampling rate of 10^8^ Hz was used with downsampling via a Cascade Integration Comb filter applying a decimation rate of 8000. The resonant frequency signal was read with a field-programmable gate array (#DE2-115, Terasic Inc.) connected via ethernet cable to a desktop computer. The experiments were performed using a custom code written in LabVIEW 2020 software, and a computer-controlled pressure regulator (#QPV1, Proportion Air) was connected to the data acquisition card (#USB-6002, National Instruments) to flow fluid and particles. The frequency shift was measured when the particle was moving back and forth through the vibrating stainless steel capillary sensor, and the custom LabVIEW program reversed the direction of fluid flow when the particle was detected.

For system validation and performance analysis, National Institute of Standards and Technology (NIST) polystyrene beads with two different diameters (400 and 500 μm, #4340A and #4350A, Thermo Fisher Scientific) were used. For bead population studies, the magnetic stir bar was placed inside the vial containing the fluid and the beads. All beads were measured in deionized (DI) water. For system cleaning following each experiment, the system was immersed in 10% (v/v) bleach for 5 min to completely wash out biological residues, after which the system was rinsed with 7% Pluronic F-127 (#P2443, Sigma-Aldrich) in DI water.

### Patient-derived GBM models

Patient-derived GBM tumor spheroids have been described previously (*48*). After enzymatic and mechanical dissociation, GBM tumor resections were seeded in NeuroCult NS-A Basal Medium (Human; #05750, STEMCELL Technologies) supplemented with additional growth factors, including epidermal growth factor (20 ng/ml), basic fibroblast growth factor (10 ng/ml), and heparin (2 mg/ml; #78006, #78003, #07980, STEMCELL Technologies). GBM tumor spheroids were passaged once every week. 1X Accutase (#AT104, Innovative Cell Technologies) was used, the tumor spheroids were incubated at 37°C to be dissociated, and single cells were resuspended in T-75 ultralow attachment flasks (#3814, Corning) at a concentration of 2000 cells/ml in 10 ml. The tumor spheroids would normally reach a suitable size for experiments in ~3 days after passage, and the spheroids were imaged using the Incucyte S3 System (Sartorius AG) to ensure appropriate spheroid diameter. Tumor spheroids were derived from the GBM model BT145. The models and more detailed information are available from the Dana-Farber Cancer Institute Center for Patient Derived Models (www.dana-farber.org/research/integrative-research/patient-derived-models).

### Preparation of media

OptiPrep medium [35% (v/v)] was made by mixing OptiPrep density gradient medium (#92339-11-2, Sigma-Aldrich) with NeuroCult medium. Phosphate-buffered saline (PBS; 1×) with heavy water was made by adding 10% (v/v) 10× PBS (#AM9624, Invitrogen) with 90% (v/v) D_2_O (#151882, Sigma-Aldrich). The media were vacuum filtered using the filter unit (#73520-990, VWR International), and their densities were measured using the Mettler Toledo Precision Balance (#ME103TE, Hogentogler & Co.) as $\rho_{Η2Ο-\text{medium}}=1037.8,\rho_{\text{OptiPrep}-\text{medium}}=1141.8,\;\text{and}\;\rho_{D2Ο-\text{medium}}=1131.3\;\text{kg}/m3$, respectively for water-based NeuroCult medium, NeuroCult medium with 35% OptiPrep, and D_2_O-based PBS.

### Staurosporine treatment of GBM tumor spheroids

For staurosporine measurement, GBM BT145 cells were seeded in 96-well round-bottom ultralow attachment plates (7007, Corning Inc., Corning, NY, USA) at a concentration of 6000 cells in a volume of 90 μl. After sufficient tumor spheroid formation, the tumor spheroids were exposed to 2 μM staurosporine (Abcam) or a control [0.1% (v/v) DMSO] for 1 hour. The tumor spheroids were measured immediately after drug exposure.

### Analysis of frequency responses

The raw data were processed using MATLAB 2019b, and the Signal Processing Toolbox was used. Savitzky-Golay filter was applied to the resonant frequency measurements from the sensor. The resulting shift Δf in resonant frequency due to a particle passing through the steel tube vibrating at the seventh resonant mode consists of seven local minima (for H_2_O and D_2_O) or maxima (for OptiPrep) altogether called antinodes, which we collectively refer to as a peak. In our signals, both from experiments and simulations, the peaks were detected by finding antinodes with prominence around 5 to 10 times higher than the SD of noise. The antinodes were identified and grouped together in sets of seven that constitute a single peak. For a given peak, its baseline resonant frequency f was defined by fitting a third-degree polynomial before and after the peak, taking for both three times the interval between the first and second antinode. Only the extreme (first and last) antinodes were used for buoyant mass determination because they have the maximum absolute shift ∣Δf∣ in resonant frequency. To smooth out the distortion of the antinodes due to noise, we fitted local second-degree polynomials to the antinodes and took the local maximum or minimum. A given peak was accepted only if the two antinode signals have a maximum of 20% relative difference. Then, the height of each peak Δf was converted to buoyant mass by

$$m_{b}=-2\;\Delta f\;m_{\text{eff}}/f/a_{m}$$ (4)

where $a_{m}$ is the mass discrepancy (*54*). The calibration of the steel tube was done by calculating $m_{\text{eff}}$ via known buoyant masses of experimental measurements of NIST polystyrene beads, assuming mean diameters from vendor. For the spheroid measurements, the effective mass $m_{\text{eff}}$ was adjusted accounting for the densities of the different fluids used.

### Determining water content

We determined cellular water content from buoyant mass measurements according to a previously established approach (*14*, *17*, *32*). We determined the total volume of the tumor spheroids by comparing buoyant mass measurements in normal medium and OptiPrep-based medium using the Eq. 1. We then determined the dry volume of the tumor spheroid by comparing tumor spheroid buoyant mass measurements in normal medium and D_2_O-based medium using Eq. 2. The absolute and fractional water content (v/v) were calculated on the basis of the dry and total volumes.

### Tumor spheroid viability measurements

The tumor spheroids were dissociated with 5-min ACCUTASE (#07920, STEMCELL Technologies) treatment and plated in a 96-well round-bottom ultralow attachment well plate (#7007, Corning) at 1500 cells/100 μl. The plate was centrifuged for 30 s at 200*g* to encourage spheroid formation at the bottom of the well. The spheroids sizes were monitored, and the spheroids were fed with 10 μl of fresh media every 3 days. The experiment was started once the spheroids reached ~500 μm in diameter. The treatment had three arms; H_2_O-based PBS, D_2_O-based PBS, and a nontreated NeuroCult medium–only control. The supernatant was carefully aspirated, leaving just the spheroid remaining, and the volume was replaced with 100 μl of the respective treatment arm. The total treatment time was 5 min, as in the buoyant mass measurements. After the treatment, the spheroids were moved back to the NeuroCult medium. Three days after treatment, the spheroid viabilities were analyzed using the CellTiter-Glo assay (Promega, #G7573).

### Determination of noise in experimental signals

The segments of the data between peaks were isolated. To characterize noise, first the low frequency (f<1) drift was removed by applying a Butterworth high-pass signal $f_{\text{high}-\text{pass}}=1\;\text{Hz}$ and order n=2. The mean of the segment was subtracted from the segment, and the SD σ of the noise was calculated in the time domain. In addition, the slow drift of the frequency baseline was characterized via the parameters of the third-degree polynomial fits applied to immediate baselines before and after each peak, taking for both three times the interval between the first and second antinode. For the characterization of the slow baseline drift, extreme outliers (<0.3% of the data) were excluded when they were over ~4 σ from the mean.

### Simulations of noise and frequency responses

The simulations were performed by taking the theoretical formula Δf for frequency change at seventh resonant mode and adding color noise with SD σ and slope a (a=0−2) of the form $S\propto 1/f^{a}$, where S is the power spectral density of the noise. For the simulations, we used σ values in the upper (>80%) percentile of the noise level observed in the experiments with tumor spheroids, and we assumed white noise (a=0). We selected these values to confidently determine an upper limit to the precision of our water content measurement. In addition, a slow baseline drift was applied to simulated signals. This drift was determined from experiments by taking normal distributions for the mean and SD of the parameters of the third-degree polynomial fits applied to the baselines before and after each peak in experiments with tumor spheroids. Because the experiments are produced by applying a PLL of second order to excite the tube at the seventh resonant mode, the experimental signal can be simplified to a first-order Butterworth low-pass filters applied to the peak and the noise (*45*). Therefore, an equivalent low-pass filter with cutoff frequency equal to the bandwidth of the PLL (80 Hz) was applied to the simulations. Data (frequency responses) were simulated for a similar number of repeat buoyant mass measurements as in the experiments, assuming the same fluid densities and the same number of tumor spheroids as in the experiments. The data from the simulations were analyzed identically to the data from the experiments.

### Statistics and data presentation

Statistical comparisons were carried out using Welch’s *t* test unless otherwise stated. All *P* values were calculated using OriginPro 2025 or MATLAB 2024b. In all box plots, the central line and square depict the median and mean, respectively; the bottom and top edges of the boxes respectively indicate the 25th and 75th percentiles; and the whiskers indicate the 5th and 95th percentiles. When only the manufacturer-provided bead reference data were converted to distributions, percentiles were defined by assuming that the data follows a normal distribution. Error propagation and statistics in water content measurements are presented in detail in note S3. Supplementary data contains the raw data used for figures.

## Supplementary Materials

### The PDF file includes:

Supplementary Notes S1 to S3 Table S1 Figs. S1 to S9 Legend for data S1
