# Supplementary material for: Inertial sensing of water content in tumor spheroids
Source: Sci Adv. 2026 Apr 3;12(14):eaeb1451. doi: 10.1126/sciadv.aeb1451 (PMC13048265; doi:10.1126/sciadv.aeb1451)
Supplement: Supplementary file 1 — Supplementary Notes S1 to S3 Table S1 Figs. S1 to S9 Legend for data S1 [file sciadv.aeb1451_sm.pdf]

Science Advances
AAAS

Supplementary Materials for

Inertial sensing of water content in tumor spheroids

Georgios Katsikis *et al.*

Corresponding author: Scott R. Manalis, srm@mit.edu; Teemu P. Miettinen, teemu@mit.edu

Sci. Adv. **12**, eaeb1451 (2026)
DOI: 10.1126/sciadv.aeb1451

The PDF file includes:

Supplementary Notes S1 to S3
Table S1
Figs. S1 to S9
Legend for data S1

Other Supplementary Material for this manuscript includes the following:

Data S1

SUPPLEMENTARY NOTE 1: Theoretical Modelling of steel tube

Calculation of normalized sensitivity

The mass-frequency relation for our resonator is:

$$\frac{\Delta f}{f} = -\frac{a_m}{2} W^2 \frac{m_b}{m_{eff}} \tag{S1}$$

where Δf is the frequency change caused by a particle of buoyant mass m_b through a tube vibrating at a resonance frequency f. W is the local amplitude of vibration at a given position of the tube and a_m is the mass discrepancy parameter[54]. The equation (S1) is the same as equation (4) in main text but expressed as a function of the frequency change relative to the baseline frequency. The effective mass m_{eff} of a hollow tube of density $\rho_{cylinder}$ containing fluid of density ρ_{fluid} is:

$$m_{eff} = \frac{\zeta L \pi}{4}\left(\rho_{cylinder}\left(d_o^2 - d_i^2\right) + \rho_{fluid} d_i^2\right) \tag{S2}$$

where for our tube's geometry, d_i and d_o are the inner and outer diameters of the cylinder, L is the length of the tube between the two clamped positions. The parameter $\zeta = \int_{x=0}^{x=1} W^2 dx$, where x is the longitudinal position on the cantilever normalized by L, expresses the equivalent amplitude of vibration for a point-mass. For the double-clamped configuration of our system, we calculated $\zeta = 0.437189$ independent of the resonant mode number. Combining equations (S1) and (S2), we derived the normalized sensitivity ($|\Delta f|/f/m_b$) with respect to particle mass at the antinodes where the frequency signal has maximum amplitude (where $W = 1$) is:

$$\frac{|\Delta f|/f}{m_b} = \frac{2a_m}{L\zeta\pi\left(\rho_{tube}\left(d_o^2 - d_i^2\right) + \rho_{fluid} d_i^2\right)} \tag{S3}$$

We selected the normalized sensitivity as a guide for the design of the tube because it is independent of the resonant mode number.

Using equation (1), we expressed the total volume as a function of the diameter and calculate an order of magnitude for average spheroids by assuming an spherical shape of diameter *d*=450 µm and total density of ρ_{total} =1,050kg/m^3 measured in pure water:

$$m_b = \frac{\pi d^3}{6}\left(\rho_{\text{total}} - \rho_{\text{fluid}}\right) \tag{S4}$$

We thus calculated buoyant mass $m_b \cong 2.5$ µg, so we focused on µg-scale particles.

To select the dimensions of our tube, we studied the variation of sensitivity in the submillimeter range ($d_i < 1$mm) while assuming a fixed thickness of the tube $d_o - d_i = 0.2$ mm (Fig. 1B). We chose this tube thickness as it represented the smallest thickness which maintained sufficient structural rigidity for screw mounting. We also

assumed that the length of the clamped portion of the cantilever should be at least an order of magnitude larger than the diameter, $L > 10\ d_i$ so that the tube can display bending resonance modes as a long slender vibrating body.

For a spheroid of a μg-scale buoyant mass, we assumed that a sensitivity of 10 ppm/μg (e.g. 0.3Hz/μg signal for baseline frequency of 30kHz) provides sufficient signal to noise for our measurements. To yield this sensitivity while allowing for practical assembly of the tube, we choose an inner diameter of d_i=600 μm, an outer diameter d_o =800 μm, a length of $L = 55$ mm (Fig. 1B). We also choose stainless steel as the material of the tube ($\rho_{tube} = 7,850\ kg/\text{m}^3$). We selected stainless steel (SUS#304) for its biocompatibility, high compressive strength (~200MPa) and commercial availability. In the context of calculation of sensitivity, we also set the mass discrepancy factor as equal to one ($a_m = 1$) because for the typical conditions of our experiments it is approximately in the range $a_m = 0.97 - 1.03$ based on the density of the particle and the solution (Materials and Methods). Using equation (S3) for the selected values, we calculated:

$$\frac{\Delta f/f}{m_b} = 10.4\frac{\text{ppm}}{\mu\text{g}}$$

Notably, the experimentally derived sensitivity of ~9 ppm/ug (Fig. 2) was within 10% of the theoretical prediction.

In a broader perspective with regards to material selection, given the same geometric parameters, using other materials such as fiberglass ($\rho_{tube} = 2{,}550\ kg/m^3$) instead of steel would yield a higher sensitivity:

$$\frac{\Delta f/f}{m_b} = 24.7\frac{ppm}{\mu g}$$

Despite the 2.5X theoretical increase in sensitivity due to the fiberglass' lower density relative to steel, we chose stainless steel to prevent local buckling at the clamping points, given the higher compressive strength of steel (200-300 MPa) compared with fiberglass (~30 MPa).

<u>Calculation of sensitivity</u>

We calculated resonant frequency of the tube as:

$$f = \frac{\lambda_n^2}{2\pi}\sqrt{\frac{EI\zeta}{m_{eff}L^3}} \tag{S5}$$

where E is Young's modulus of material of tube, $I = \pi/64(d_o^4 - d_i^4)$ is moment of inertia and $\lambda_n = [4.73, 7.85, 11.0, 14.1, 17.3, 20.4, 23.6, 26.7, 29.8]$ are eigenvalues with three-digit precision for first eight resonant modes and m_{eff} is given by equation (S2). By putting the selected dimensions and material properties in equation (S5), we predicted that the resonant frequencies for 4th to 7th modes are in the kHz range (Fig. S1B).

Multiplying equations (S3) and (S5), we derived the sensitivity $s = |\Delta f|/m_b$ of the tube as:

$$s = \frac{a_m \lambda_n^2}{\pi} \sqrt{\frac{EI\zeta}{m_{eff}^{5/2} L^3}} \tag{S6}$$

The sensitivity is also calculated experimentally by measuring the frequency change Δf caused by flowing particles of known buoyant mass (i.e. known distribution with mean) and known fluid densities from equations (S1) and (S4). Here, we theoretically calculated the sensitivities for various fluids as $s_{H2O-media} = 0.34194\ \mathrm{Hz/ug}$, $s_{Optiprep-media} = 0.33461\ \mathrm{Hz/ug}$ and $s_{D2O-media} = 0.33534\ \mathrm{Hz/ug}$ respectively for experimentally-derived fluid densities $\rho_H = 1037.8\ \mathrm{kg/m3}$, $\rho_O = 1141.8\ \mathrm{kg/m3}$ and $\rho_D = 1131.3\ \mathrm{kg/m3}$.

Since the sensitivity is used to convert change in frequency to buoyant mass, we will interchangeably use the term calibration factor for s.

SUPPLEMENTARY NOTE 2: System calibration and its influence on water content determination

System accuracy

The buoyant mass measurement accuracy of our steel tube system was determined by calibrating the system using NIST-traceable polystyrene beads and then examining the measurement accuracy on a separate day using separate beads (Fig. S8). This revealed that our system accuracy was approximately 90%. Notably, our calibration and verification experiments were carried out approximately a month apart, and the small accuracy bias may reflect a calibration drift over time. Calibration drift may occur due to accidental tampering with device or stress relaxation on the thin tube.

Calibration influence on water content determination

The determination of total density, dry density, and fractional water content are largely insensitive to uncertainties in the calibration of the steel tube, because the calculation of these metrics depends only on the ratio of the particle's buoyant masses in different fluids, thus cancelling out the calibration. However, the calibration within each fluid can differ slightly. To examine the degree to which the fluid-specific calibration can influence our key metric, the fractional water content, r_{w}, we calculated the theoretical influence of fluid density on the calibration factor. Using these theoretical values, we then examined how the fractional water content of our tumor spheroids would be influenced given fluid-specific calibration factor variation (Fig. S9). This revealed that in our measurements the calibration factors are very similar, resulting in a maximum fractional water content, r_{w}, accuracy error of ~1% due to variation in the calibration for different fluids.

SUPPLEMENTARY NOTE 3: Error propagation and statistical analysis

<u>Calculating population-level water content</u>

Let m_1, m_2, and m_3 be a particle's mean buoyant masses in H_2O, OptiPrep, and H_2O-based media respectively and ρ_1, ρ_2 and ρ_3 are the densities of each respective measurement medium.

First to derive a particle's total volume, we used equation (1) for the measurements of the particle's buoyant masses in H_2O and OptiPrep media:

$$V_{total} = -\frac{m_1 - m_2}{\rho_1 - \rho_2} \tag{S7}$$

Similarly, to derive a particle's dry volume, we used equation (2) for the measurements of the particle's buoyant masses in H_2O and D_2O media:

$$V_{dry} = -\frac{m_1 - m_3}{\rho_1 - \rho_3} \tag{S8}$$

Substituting equations (S7) and (S8) in equation (3), we derived fractional water content r_W as a direct function of the experimentally measured buoyant masses:

$$r_w = \frac{m_1(\rho_2 - \rho_3) + m_2(\rho_3 - \rho_1) + m_3(\rho_1 - \rho_2)}{(m_1 - m_2)(\rho_1 - \rho_3)} \tag{S9}$$

<u>Calculating population-level water content</u>

Each buoyant mass from equation (S9) is connected to a frequency change via sensitivity of calibration factor either defined experimentally or calculated theoretically from equation (S7) as:

$$m_i = \Delta f_i / s_i,\ i = 1,2,3 \tag{S10}$$

Therefore, by combining equations (S9) and (S10), we can derive the fractional water content as a function only of ratio of calibration factors:

$$r_w = \frac{\Delta f_1(\rho_2 - \rho_3) + s_{12}\Delta f_2(\rho_3 - \rho_1) + s_{13}\Delta f_3(\rho_1 - \rho_2)}{(\Delta f_1 - s_{12}\Delta f_2)(\rho_1 - \rho_3)} \tag{S11}$$

where $s_{ij} = s_i/s_j$, $i,j = 1,2,3$. Using the theoretical values for calibration factors from the previous section, and the mean frequency changes from experiments, we examined the impact of s_{ij} on r_w (Fig. S9). Compared with r_w assuming the calibration factors in all fluids are equal to the value in water (i.e. $s_{12} = s_{13} = 1$), the maximum

variation in r_{w} is approximately ±1%, implying that the assumption that $s_{12} = s_{13} = 1$ is reasonable and that we can assume that the calibration factors in all fluids are equal.

Error propagation for water content measurements

Next, we calculated the error δr_{w} in the fractional water content based on the standard errors (SEM) of the buoyant masses in the three media types ($\delta m_1, \delta m_2, \delta m_3$). The SEM of the buoyant masses are:

$$\delta m_1 = \frac{\sigma_1}{\sqrt{n_1}}, \delta m_2 = \frac{\sigma_2}{\sqrt{n_2}}, \delta m_3 = \frac{\sigma_3}{\sqrt{n_3}} \tag{S12-S14}$$

where $\sigma_1, \sigma_2, \sigma_3, n_1, n_2,$and n_3 are the standard deviations and sample sizes of buoyant mass measurements performed in each respective measurement medium. Propagated fractional water content error is:

$$\delta r_{\mathrm{w}} = \sqrt{\sum_{k\in\{1,2,3\}} \left(\frac{\partial r_{\mathrm{w}}}{\partial m_k} \delta m_k\right)^2} \tag{S15}$$

Then, we calculated partial derivatives with respect to buoyant masses in each fluid from equation (S15):

$$\frac{\partial r_{\mathrm{w}}}{\partial m_1} = \frac{(m_2 - m_3)(\rho_1 - \rho_2)}{(m_1 - m_2)^2(\rho_1 - \rho_3)} \tag{S16}$$

$$\frac{\partial r_{\mathrm{w}}}{\partial m_2} = -\frac{(m_1 - m_3)(\rho_1 - \rho_2)}{(m_1 - m_2)^2(\rho_1 - \rho_3)} \tag{S17}$$

$$\frac{\partial r_{\mathrm{w}}}{\partial m_2} = \frac{\rho_1 - \rho_2}{(m_1 - m_2)(\rho_1 - \rho_3)} \tag{S18}$$

Hence, we calculated the propagated error of fractional water content by combining equations (S15) to (S18):

$$\delta r_{\mathrm{w}} = \sqrt{\frac{\delta m_3^2(\rho_1 - \rho_2)^2}{(m_1 - m_2)^2(\rho_1 - \rho_3)^2} + \frac{\delta m_1^2(m_2 - m_3)^2(\rho_1 - \rho_2)^2}{(m_1 - m_2)^4(\rho_1 - \rho_3)^2} + \frac{\delta m_2^2(m_1 - m_3)^2(\rho_1 - \rho_2)^2}{(m_1 - m_2)^4(\rho_1 - \rho_3)^2}} \tag{S19}$$

Statistical tests for water content comparisons—Welch-Satterthwaite T test

To perform pairwise statistical comparisons of spheroid fractional water contents, we used a two-sided Welch's T test, using the Welch-Satterthwaite equation to estimate the equivalent degrees of freedom of each population-level fractional water content value. The null hypothesis for this test is that the two spheroids in question have the same mean fractional water content value, and the alternative hypothesis is that the two spheroids have different mean water contents.

Given two spheroids A and B with average fractional water contents $r_{\mathrm{w,A}}$ and $r_{\mathrm{w,B}}$ and errors $\delta r_{\mathrm{w,A}}$ and $\delta r_{\mathrm{w,B}}$, we can calculate the t-statistic:

$$\mathrm{t} = \frac{r_{\mathrm{w,A}} - r_{\mathrm{w,B}}}{\sqrt{\delta r_{w,A}^2 + \delta r_{w,B}^2}} \tag{S20}$$

To calculate the Welch-Satterthwaite degrees of freedom, we first need the effective degrees of freedom ν_A and ν_B respectively for each spheroid. Each of these is a function of the SEM $(\delta m_1, \delta m_2, \delta m_3)$ and sample size (n_1, n_2, n_3) of buoyant mass measurements in each fluid. We calculate ν_A and ν_B:

$$\nu_A \approx \frac{\sum_{k\in\{1,2,3\}} u_k^2}{\sum_{k\in\{1,2,3\}} \frac{u_k^4}{\nu_k}} \tag{S21}$$

$$\nu_B \approx \frac{\sum_{k\in\{1,2,3\}} u_k^2}{\sum_{k\in\{1,2,3\}} \frac{u_k^4}{\nu_k}} \tag{S22}$$

where the degrees of freedom for buoyant mass measurements in each fluid $\mathrm{\nu_k = n_k - 1}$ and the variance contributed to the water content measurement from buoyant measurements in fluid k is $u_k^2 = \left(\frac{\partial r_{\mathrm{w,i}}}{\partial m_k}\right)^2 \delta m_k^2$ where I is spheroid A or B. Using equations (S17) and (S18), we can calculate the total equivalent degree of freedom ν:

$$\nu = \frac{\left(\delta r_{w,A}^2 + \delta r_{w,B}^2\right)^2}{\frac{\delta r_{w,A}^4}{\nu_A} + \frac{\delta r_{w,B}^4}{\nu_B}} \tag{S23}$$

Finally, we can calculate a p-value from the t-distribution:

$$p = 2\left(1 - F_T(\nu, t)\right) \tag{S24}$$

where F_{T_ν} is the cumulative density function of the T-distribution. Practically, comparing the spheroids with fractional water contents of 93.23% and 91.97% (Fig. 4D), we find spheroid-specific degrees of freedom $\nu_1 = 64.98$ and $\nu_2 = 77.23$ and a total equivalent degree of freedom $\nu = 139.21$. With a t-statistic of $t = 2.72$, we compute a p-value of 0.0074. We therefore reject the null hypothesis, concluding that these two spheroids have different mean water contents. P-values from the comparison of the spheroid with 93.23% fractional water content with each of the other experimentally measured spheroids are listed below (Table S1).

Welch-Satterthwaite p-value (compared with $r_{\mathrm{w}} = 93.23\%$ spheroid)
7.405×10^{-3}
1.231×10^{-6}
3.126×10^{-7}
8.457×10^{-9}
1.996×10^{-5}
4.308×10^{-5}
6.474×10^{-2}
1.927×10^{-12}
9.855×10^{-9}
6.883×10^{-15}

Table S1. Welch-Satterthwaite p-values from the comparison of the spheroid with 93.23% fractional water content with that of each other experimentally measured spheroid.

With significance level $a = 0.05$, since all $p < a$, the water content of the spheroid with $r_{\mathrm{w}} = 93.23\%$ is different from all other spheroids, except one. We conclude that the tumor spheroid population as a whole has heterogeneous water content. Equivalently, this degree of statistical significance across measured spheroids implies that our measurement precision is sufficient to resolve inter-spheroid biological water content variability.

Statistical tests for water content comparisons—Weighted-residual chi-squared test

To determine statistically if the fractional water content of all the tumor spheroids is the same, given the propagated error for each value, we used a weighted-residual chi-squared test. The null hypothesis for this test is that all spheroid water contents are equal to the mean water content value across the spheroids. The alternative hypothesis is that not all spheroid water contents are equal.

Given a single-spheroid fractional water content $r_{w,i}$, associated propagated SEM $\delta r_{w,i}$, and mean fractional water content across n spheroids $\overline{r_w}$:

$$\chi^2 = \sum_{i=1}^{n} \frac{\left(r_{w,i} - \overline{r_w}\right)^2}{\delta r_{w,i}^2} \tag{S25}$$

The associated p-value is:

$$p = 1 - F_{\chi^2}(\nu, t) \tag{S26}$$

where F_{χ^2} is the cumulative distribution function for the chi-squared distribution. For the experimental data with $\overline{r_w}$=0.912 and propagated errors (Fig. 4D), this evaluates to $\chi^2 = 155.91$ and p=2.259× 10^{-28}. Given that this p-value is less than the $\alpha = 0.05$ significance threshold, we reject the null hypothesis and conclude that not all the spheroid water content values are equal.

SUPPLEMENTARY FIGURES

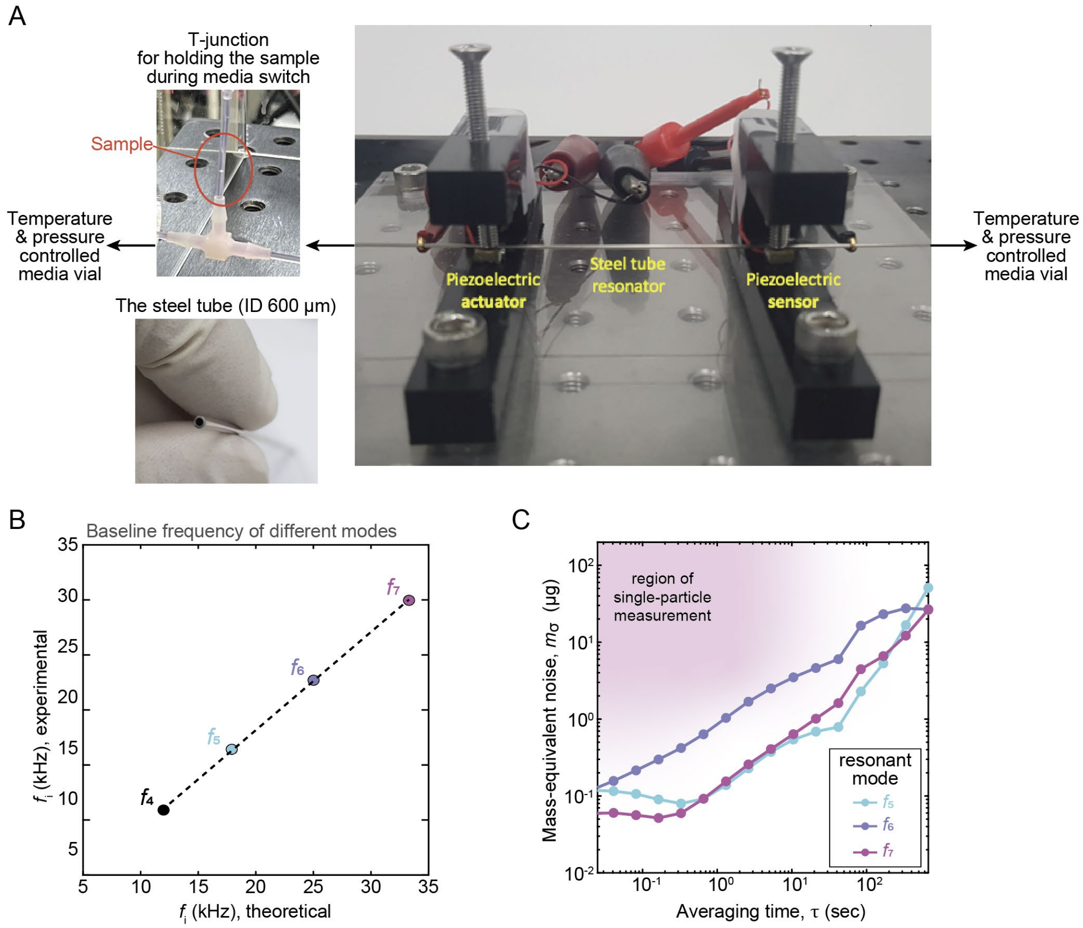

Figure S1. Details of the steel tube-based measurement setup.

(A) Images of the steel tube-based measurement setup. At each end, the steel tube is connected to tubing leading to temperature and pressure-controlled medium vials. These pressure controls are used to adjust the fluid flow in the steel tube. On one side, the connecting tubing contains a T-junction with a syringe, which can be used to temporarily hold the measured sample in a small and separate volume, as the measurement fluids in the source vials and the steel tube are changed. The bottom left displays an image of the steel tube when held separately. **(B)** Correlation between the theoretical and experimental frequencies of each resonant mode. **(C)** Allan deviation, expressed as mass-equivalent noise, of time-series data of baseline resonant frequencies.

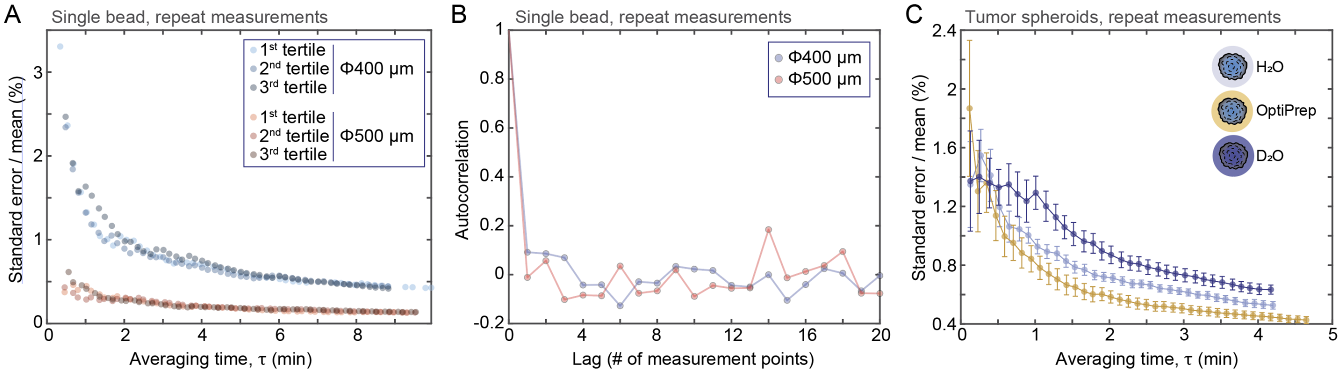

Figure S2. Characterization of buoyant mass measurement precision.

(A) Repeated measurement precision (standard error of mean, normalized to %) as a function of measurement acquisition time for 500 and 400 µm polystyrene particles. Data is the same as in Fig. 2D, but each trace is separated into three sections to exemplify the stability of the measurements. **(B)** Autocorrelations of repeated measurements of 500 and 400 µm polystyrene particles. **(C)** Repeated measurement precision (standard error of mean, normalized to %) as a function of measurement acquisition time for GBM tumor spheroids in three different fluids. Data depicts mean and SEM of 11 separate tumor spheroids.

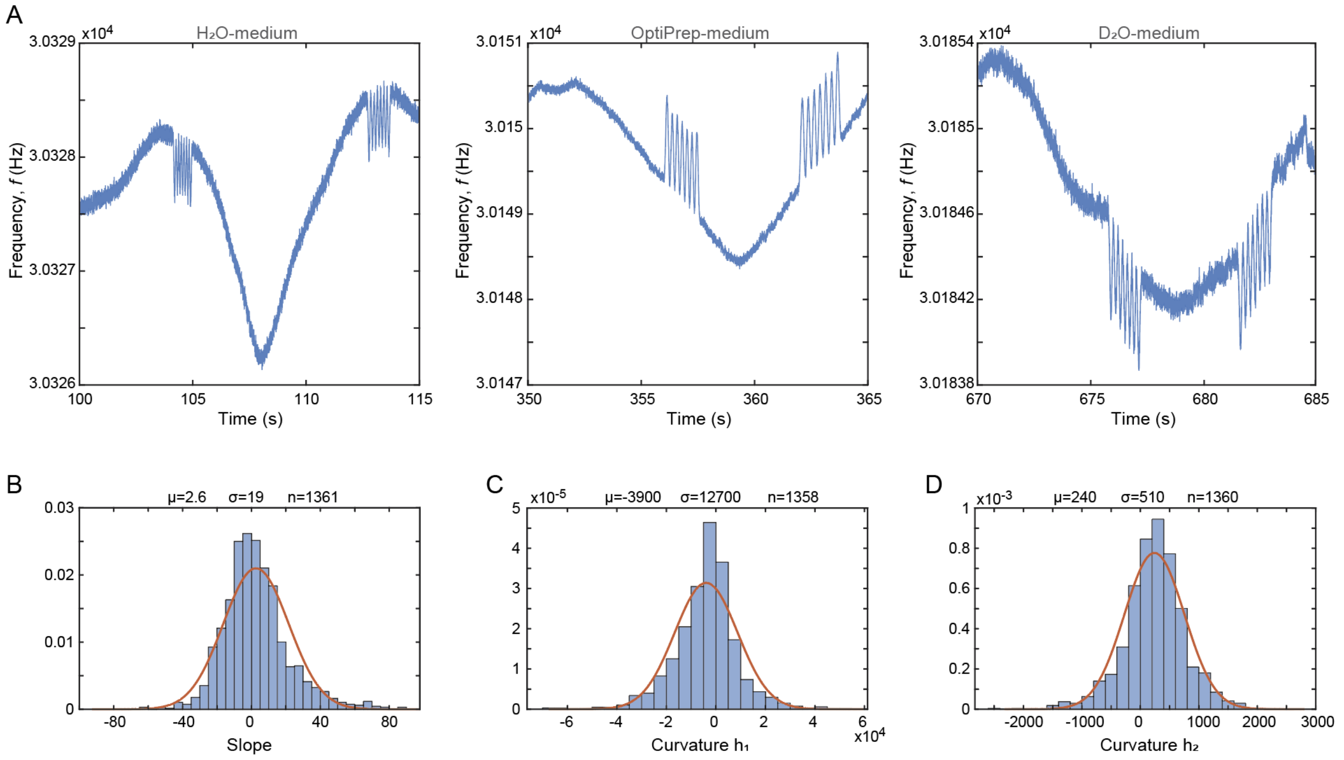

Figure S3. Buoyant mass measurements in multiple media contain baseline fluctuations.
(A) Representative raw data of GBM tumor spheroid measurements in three different media. As a small volume of the fluids is carried over when the fluids are changed, the incomplete mixing of the fluids results in baseline fluctuations. Note that the y-axis values are relative to a reference value and cannot be compared between plots. **(B-D)** Quantification of the low frequency ($f < 1\ Hz$) drift of the baseline. Each histogram depicts a separate parameter of the 3rd degree polynomial fits applied to immediate baselines before and after each peak. Curvature h_1 refers to the 2nd order curvature, and curvature h_2 refers to the 3rd order curvature. μ, σ, n refer to mean, standard deviation, and number of data points, respectively.

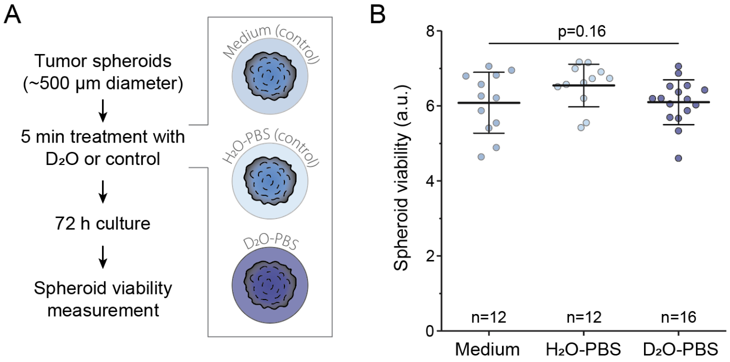

Figure S4. Evaluation of short D_2O exposure toxicity.

(A) Schematic of experimental setup. Tumor spheroids were exposed to one of three conditions (normal culture medium, PBS made into normal water, and PBS made into heavy water) for 5 minutes to mimic the exposure during buoyant mass measurements. The spheroids were then washed and moved to normal culture medium for 3 days, after which spheroid viability was examined. **(B)** Measurements of tumor spheroid viability 3 days after control or heavy water exposure. P-value was determined using ANOVA. Each dot depicts a separate tumor spheroid. Bar and whiskers depict mean±std.

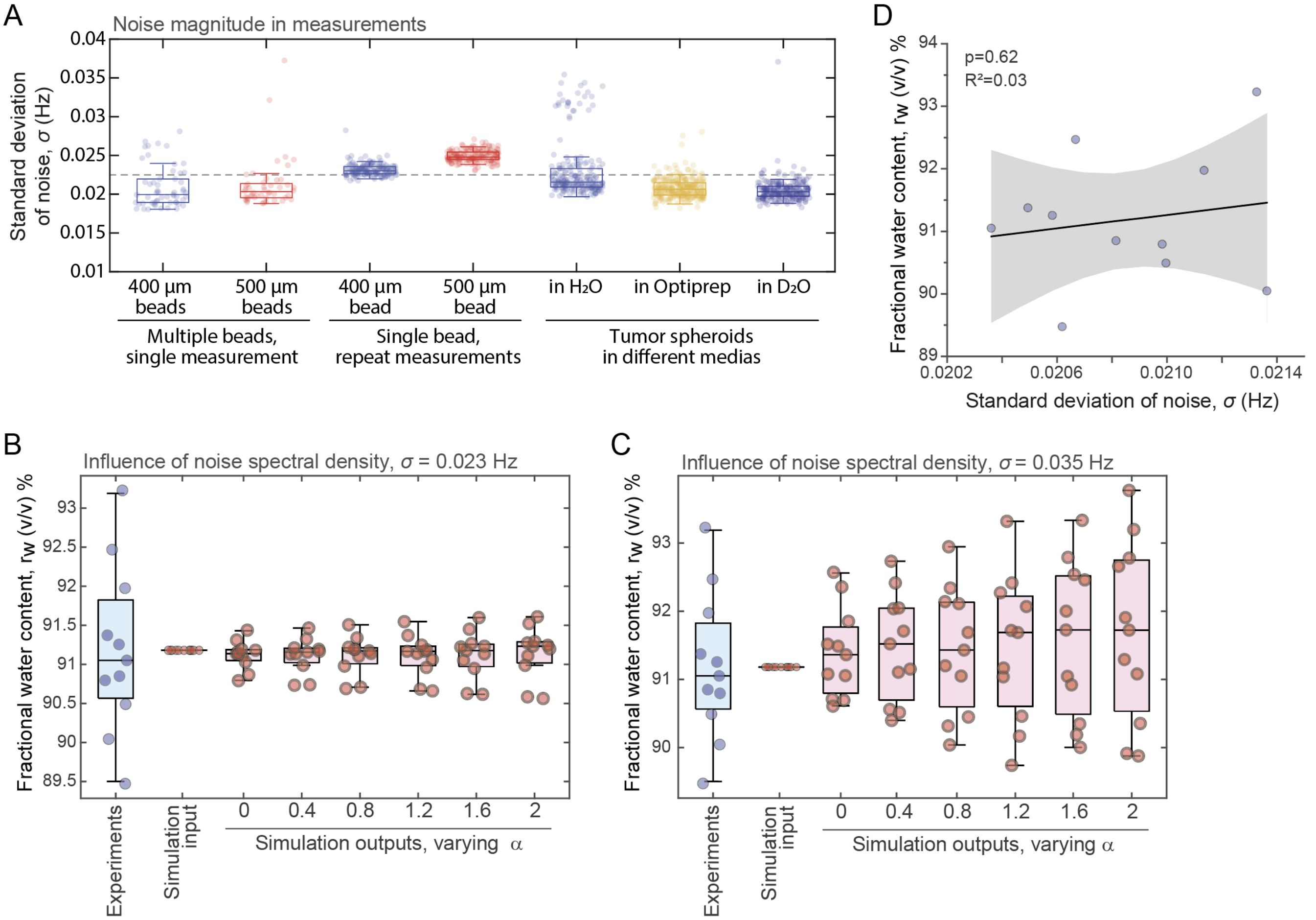

Figure S5. Evaluation of water content measurement noise in experimental and simulated datasets.
(A) Baseline noise magnitude (σ) from indicated experiments/experimental conditions. On average across all data, we observed that σ~0.0225 Hz (dashed horizontal line). Each opaque dot depicts a baseline section between two buoyant mass measurements. **(B-C)** Fractional water content, r_w, in experimental and simulated data. Simulations were run with indicated noise spectral density slope values (α) using either low (B) or high (C) noise magnitude (σ). The lower noise magnitude (panel B) is similar to the noise observed in tumor spheroid experiments. Each dot depicts a separate tumor spheroid (n=11). **(D)** Correlation between baseline noise magnitude (σ) and the derived fractional water content, r_w, in experiments. Each dot depicts a separate tumor spheroid (n=11). Black line and shaded area depict a linear fit and 95% confidence bands.

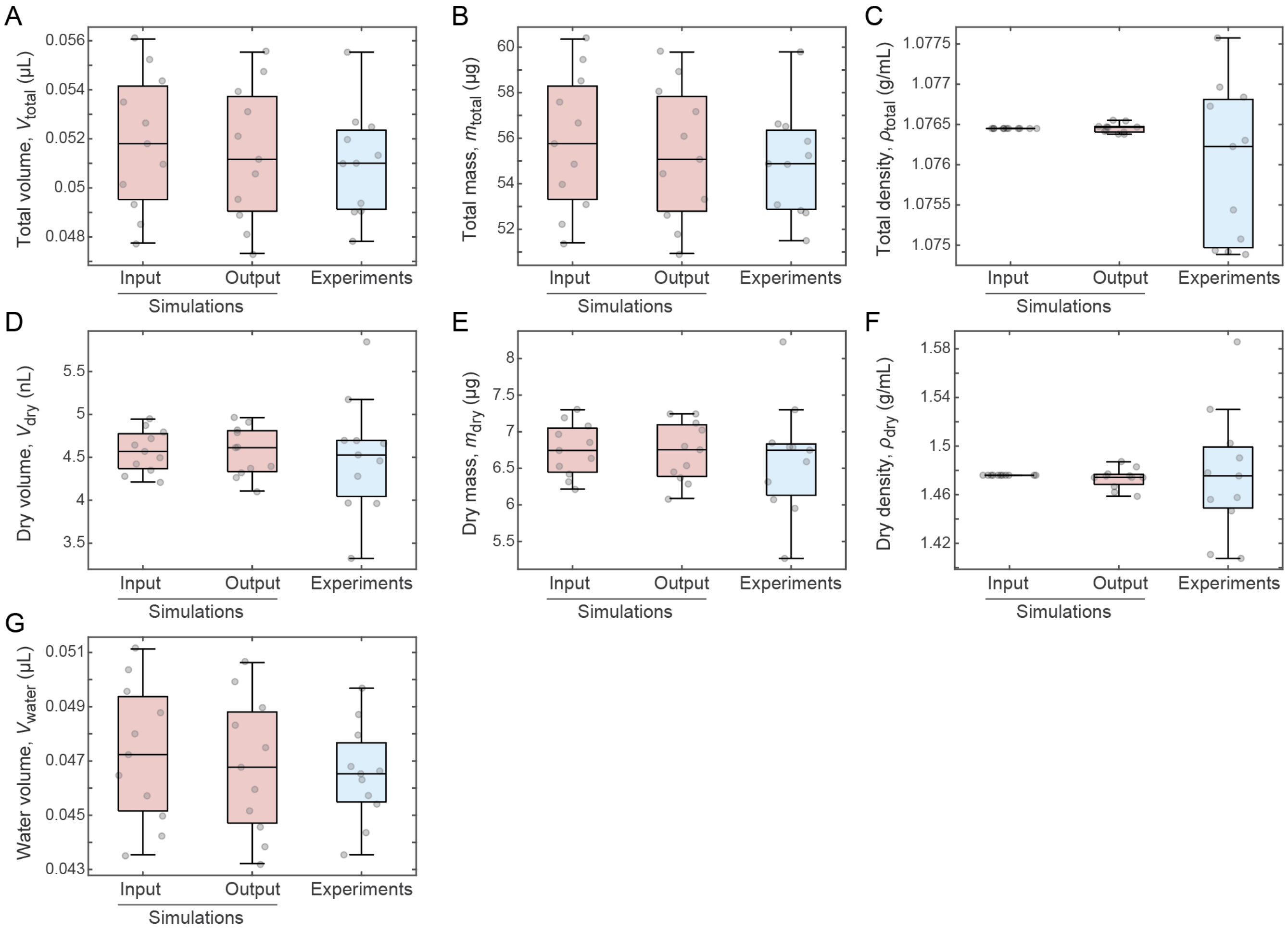

Figure S6. Simulated and measured biophysical features of GBM tumor spheroids.

(A-G) Simulated (input and output, red) and experimentally determined (blue) GBM tumor spheroid total volumes (A), total masses (B), total densities (C), dry volumes (D), dry masses (E), dry densities (F), and water volumes (G). Each opaque dot depicts a separate tumor spheroid (n=11).

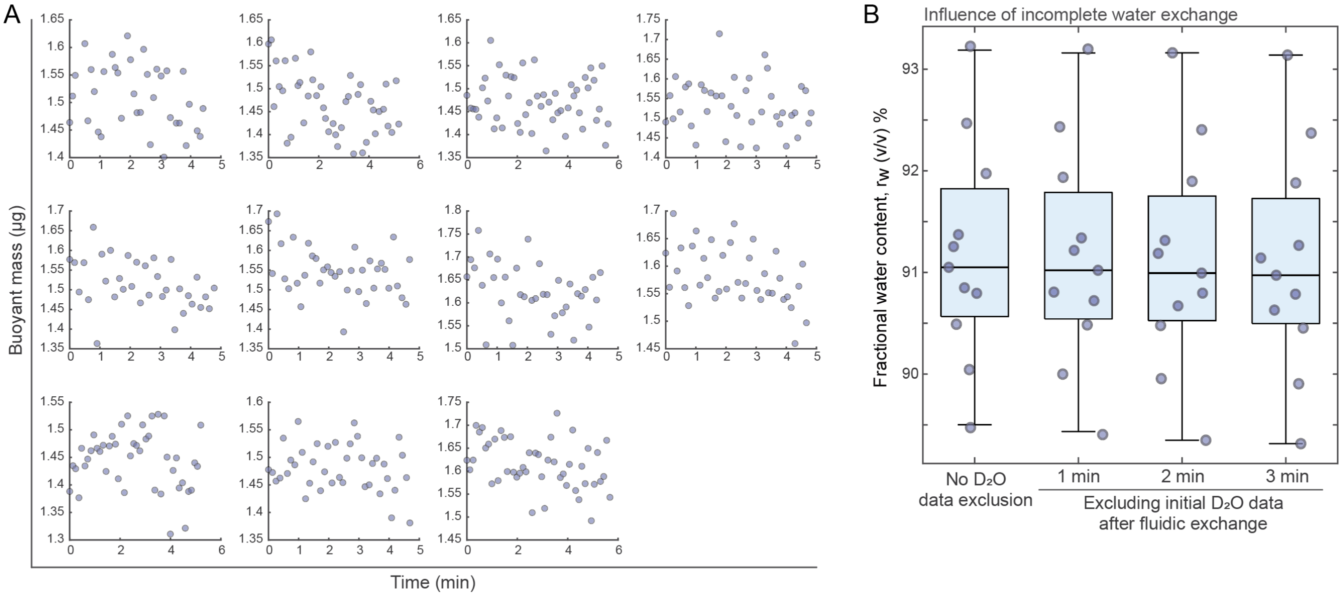

Figure S7. Evaluation of water exchange completeness.

(A) Correlation between buoyant mass and measurement time in repeated measurements of individual tumor spheroids in D_2O-based medium. Each subplot depicts a separate tumor spheroid (n=11). **(B)** Fractional water content, r_w, when excluding initial buoyant mass measurements in the D_2O-based medium. The calculated water content changes only 0.11% when excluding the first 3 minutes of buoyant mass measurements in D_2O-based medium. This change is within our measurement error of ~0.5% (Fig. 4D).

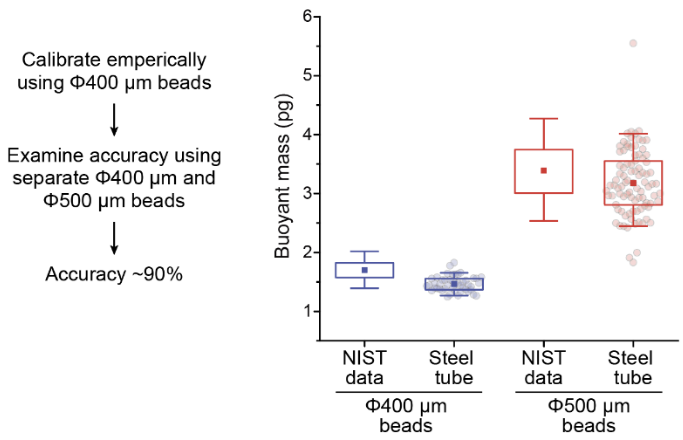

Figure S8. Examination of accuracy of steel tube using calibration beads.

Our system's buoyant mass measurement accuracy was examined by first calibrating the system using 400 µm diameter polystyrene beads, after which the accuracy was tested with a measurement of 400 µm and 500 µm diameter beads of known sizes (*right*). 'NIST data' refers to supplier provided size and 'Steel tube' refers to our measurements. Individual dots depict single beads (n = 47 and 90). The calibration and the follow-up accuracy tests were carried out approximately one month apart.

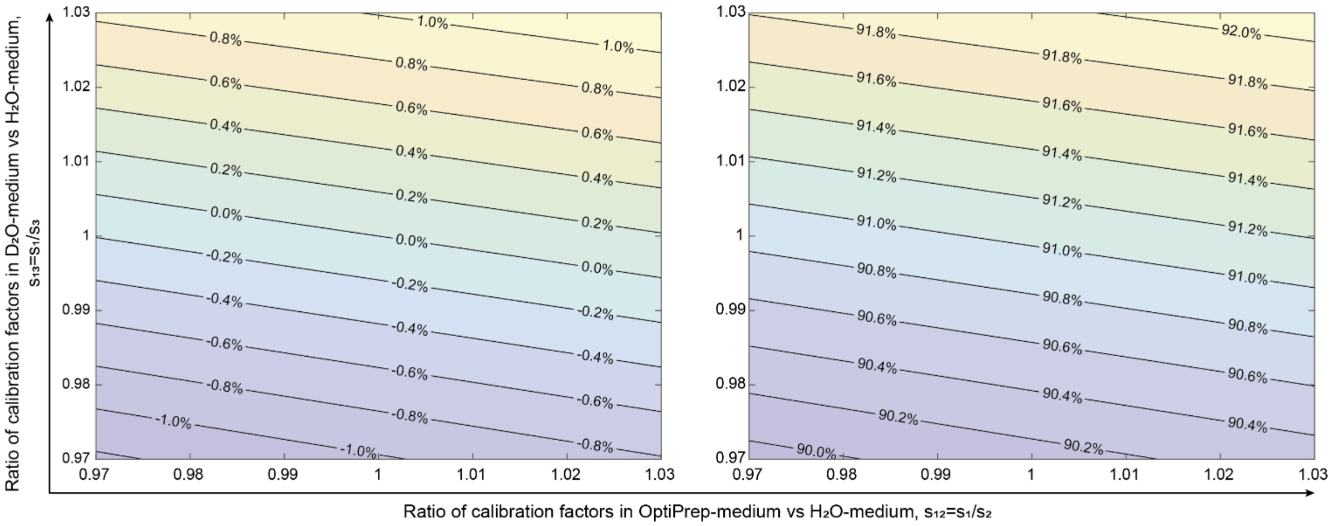

Figure S9. Influence of fluid-specific calibration to the determination of fractional water content. (*Right*) Contour plot of fractional water content, r_{w}, as a function of ratios of buoyant mass calibration factors for OptiPrep-media and D_2O-media relative to the calibration factor in H_2O-media. r_{w} values, calculated using equation (S11) are taken as an average over experimental spheroid measurements. (*Left*) Contour plot of percent difference of fractional water content $\Delta r_{\mathrm{w}} = (r_{\mathrm{w}} - r_{\mathrm{w}}|_{s_{12},s_{13}=1})/\ ((r_{\mathrm{w}} + r_{\mathrm{w}}|_{s_{12},s_{13}=1})/2)$, relative to the assumption that calibration factors are the same in all fluids ($r_{\mathrm{w}}|_{s_{12},s_{13}=1}$). For both plots, we theoretically calculated the sensitivities for various fluids as $s_1 = s_{H2O-media} = 0.34194\ \mathrm{Hz/ug}$, $s_2 = s_{Optiprep-media} = 0.33461\ \mathrm{Hz/ug}$ and $s_3 = s_{D2O-media} = 0.33534\ \mathrm{Hz/ug}$ respectively for fluid densities $\rho_H = 1037.8\ \mathrm{kg/m3}$, $\rho_O = 1141.8\ \mathrm{kg/m3}$ and $\rho_D = 1131.3\ \mathrm{kg/m3}$ using equation (S6). The limits on the axes represent an overestimate of the theoretically determined deviations of s_2 and s_3 from s_1 (using theoretical values, $s_{12} = 1.022$ and $s_{13} = 1.020$).

SUPPLEMENTARY DATA CAPTION

Data S1

Data S1 contains 29 datasheets of numerical data used to create each figure. Datasheet name indicates the figure and panel that the data was used for. Each datasheet column contains a title detailing the data metric.
